# Editorial: Exploring adaptive immunity in ruminants and humans: a one health perspective

**DOI:** 10.3389/fimmu.2026.1836173

**Published:** 2026-04-16

**Authors:** Zhipeng Zhang, Jie Cheng, Yi Yang

**Affiliations:** 1 College of Animal Science and Technology, Yangzhou University, Yangzhou, China; 2 Jiangsu Co-innovation Center for Prevention and Control of Important Animal Infectious Diseases and Zoonoses; College of Veterinary Medicine, Yangzhou University, Yangzhou, China; 3 College of Biotechnology, Jiangsu University of Science and Technology, Zhenjiang, China

**Keywords:** adaptive immunity, heat stress, mRNA, one health, ruminants, T cells, TLR4 signaling, vaccine

The intricate tapestry of life on our planet is woven with threads of interconnectedness, a reality starkly illuminated by the field of immunology. The “One Health” concept, which recognizes the inextricable link between human, animal, and environmental health, has become an operational necessity in addressing health challenges that transcend species boundaries. This is particularly evident in the study of adaptive immunity, where the mechanisms that protect us from disease are mirrored, yet uniquely adapted, in the animals with whom we share our world. This Research Topic, *Exploring Adaptive Immunity in Ruminants and Humans: A One Health Perspective*, was conceived to explore these parallels and divergences, focusing on ruminants—cattle and sheep—as critical sentinel species and partners in human society. Over the past months, we have assembled a collection of six groundbreaking studies that collectively advance our understanding of host defense, from the cellular dynamics of infection to the physiological impacts of a changing climate and the molecular evolution of immune pathways.

## 1 Deciphering host-pathogen interactions and immune cell dynamics

A core theme of this Research Topic is the detailed characterization of adaptive immune responses to specific pathogens and their underlying cellular machinery. In a pioneering study, Yu et al. delve into the often-overlooked realm of subclinical mastitis caused by Staphylococcus chromogenes. Their work provides the first evidence that γδ T cells are not merely bystanders but active participants in the immune defense of the bovine mammary gland during this chronic infection. The expansion of γδ T cells expressing CD44 and their robust production of IFN-γ, IL-17, and cytotoxic molecules (GZMB) reveal a critical, first-line role for these unconventional lymphocytes. Simultaneously, the observed reduction in CD4^+^CD8^+^ double-positive T cells in the milk points to a potential immunological vulnerability, suggesting a state of exhaustion that may predispose animals to more severe, clinical mastitis. Beyond veterinary applications, these findings have implications for human health, as Staphylococcus species are also important human pathogens. The characterization of γδ T cell responses in the bovine mammary gland may inform our understanding of similar immune dynamics in human mucosal tissues, such as the gastrointestinal tract and skin, where γδ T cells serve analogous barrier surveillance functions.

Complementing this cellular focus, Jia et al. present a paradigm shift in vaccine development against Clostridium perfringens, a significant pathogen for both livestock and humans. Their development of a lipid nanoparticle (LNP)-encapsulated mRNA vaccine targeting the conserved C-terminal domain of alpha-toxin (CPA-CTD) represents a leap forward from traditional toxoid and subunit vaccines. The vaccine’s ability to induce potent, rapid humoral and cellular immunity in both mice and cattle—the target species—is remarkable. Crucially, their demonstration of efficient passive immunity transfer from vaccinated pregnant cows to their newborn calves via colostrum addresses a critical vulnerability in neonatal livestock health. This study not only offers a powerful new tool for veterinary medicine but also serves as a compelling model for mRNA vaccine platforms against bacterial pathogens that threaten human and animal health alike.

## 2 Unveiling the impact of environmental stress on immunity

The health of both humans and ruminants is profoundly influenced by our shared environment, and climate change is a dominant stressor. Two studies in this topic powerfully illustrate how heat stress … reprograms the immune landscape of dairy cows, with implications for animal welfare, agricultural productivity, and potentially human health. In a meticulously controlled study, Koch et al. track the dynamic immune and molecular responses in blood and peripheral blood mononuclear cells (PBMCs) of cows subjected to chronic heat stress. Their work reveals a complex picture: heat stress triggers transient activation of the leukocytic NF-κB p65 pathway, likely in response to increased gut permeability and endotoxin translocation, while simultaneously suppressing T cell receptor signaling. This period of immune activation followed by suppression may explain the heightened susceptibility to infections like mastitis in heat-stressed animals. From an economic perspective, this translates into increased veterinary costs, reduced milk production, and premature culling—losses that ultimately affect food security and consumer prices. Importantly, the findings from this bovine model may inform human heat stress research, as both species share conserved physiological responses to hyperthermia. For instance, the observed activation of coagulation pathways in heat-stressed cows suggests that clinicians should monitor coagulopathy in human patients presenting with heat-related illnesses, a consideration that is currently underappreciated.

Expanding on this theme, Ye et al. provide a comprehensive review that synthesizes the field’s understanding of how heat stress impacts the bovine immune system. They present a compelling case for moving beyond traditional environmental indicators like the Temperature-Humidity Index (THI) towards a new generation of immune-related biomarkers. By highlighting the dynamic changes in innate and adaptive immunity—from compromised gut barriers and altered neutrophil function to shifts in T-cell subsets and cytokine profiles—the authors argue for a personalized approach to heat stress management. Biomarkers such as the Neutrophil-to-Lymphocyte Ratio (NLR), Heat Shock Proteins (HSPs), and specific cytokine profiles offer a window into the individual animal’s physiological state, enabling early intervention and guiding the selection of more resilient, heat-tolerant lineages. This review elegantly connects environmental stress to immunological outcome and provides a practical roadmap for enhancing the climate resilience of the dairy industry.

## 3 From evolution to function: a deeper look at macrophage and TLR4 biology

A true One Health perspective requires not only understanding present-day responses but also appreciating the evolutionary history that shaped them. Hecker et al. address a fundamental gap in our understanding of macrophage biology in ruminants. Their comprehensive characterization of ovine monocyte-derived macrophages (oMOs) polarized with M-CSF (M-oMOs) versus GM-CSF (GM-oMOs) provides a refined framework for selecting appropriate macrophage models in veterinary immunology. Using transcriptomics and functional assays, they demonstrate that GM-oMOs are terminally differentiated pro-inflammatory cells, while M-oMOs exist in a more plastic, regulatory state capable of shifting phenotype upon stimulation. This distinction is critical for designing *in vitro* models of host-pathogen interaction: M-oMOs, with their ability to dynamically respond to stimuli, more accurately recapitulate the *in vivo* immune environment during infection. By providing a validated, species-specific model system, this work enables more accurate mechanistic studies of ovine infectious diseases, which in turn can yield insights applicable to both livestock health and comparative studies of human macrophage biology.

Finally, taking the longest view, Verma and Sowdhamini delve into the deep evolutionary history of the TLR4 signaling pathway—a cornerstone of innate immunity that bridges to adaptive responses. By modeling the TIR domains of TRAM and TRIF adaptor proteins across diverse taxa, from the ancient ghost shark to humans, they reconstruct the evolutionary trajectory of this critical pathway. Their work suggests that the functional TRAM-TRIF complex, which is responsible for the MyD88-independent arm of TLR4 signaling, may have emerged later in evolution. In ancient species like Callorhinus milli, the presence of TRAM and TRIF with key residues and motifs in TRIF, but not in TRAM, implies a “work in progress.” This suggests that the MyD88-dependent pathway or TLR3-TRIF-mediated signaling may have been the ancestral mechanisms. By mapping the structural and energetic constraints that led to the fully functional pathway in mammals, this study provides profound insights into how immune systems across vertebrates—not merely our own— were built layer by layer over evolutionary time.

## 4 Conclusion: a collective step forward

The six studies assembled in this Research Topic illuminate the adaptive immune system of ruminants as a complex and dynamic entity, one that is shaped by evolutionary history, responsive to environmental stressors, and intricately involved in host-pathogen interactions. Detailed host-pathogen studies in dairy cattle can uncover mechanisms of adaptive immunity both specific to ruminants and widely shared with humans that can inform the health of multiple species. Insights from bovine heat stress models offer translatable lessons for understanding and managing the effects of climate change on human immune function. Evolutionary analyses of immune pathways across vertebrates reveal the deep commonalities that unite all mammalian species. By fostering cross-species dialogue, the One Health approach enables us to translate discoveries from ruminant immunology into strategies that benefit both animal and human health. We extend our gratitude to all authors, reviewers, and editors who contributed to this collection. Their work represents a significant stride towards a more integrated, resilient, and healthier future for all species on our shared planet.

